# Evidence from mendelian randomization identifies several causal relationships between primary membranous nephropathy and gut microbiota

**DOI:** 10.1080/0886022X.2024.2349136

**Published:** 2024-05-21

**Authors:** Jianwei Wu, Jing Zhang, Gang Huang, Yinglian Zhong, Yi Yang, Peng Deng

**Affiliations:** a Department of Medical Technology, Gannan Healthcare Vocational College, Ganzhou, China; b Department of Laboratory, GanZhou Cancer Hospital, Ganzhou, China; c Department of Blood Transfusion, Ganzhou Fifth People’s Hospital, Ganzhou, China; d Department of Rheumatology and Immunology, The Second Affiliated Hospital of Nanchang University, Nanchang, China; e Department of Endocrinology, Department of Nephrology, Ganzhou Fifth People’s Hospital, Ganzhou, China

**Keywords:** Gut microbiota, primary membranous nephropathy, mendelian randomization, genome-wide association

## Abstract

**Background:**

Research has showcased a correlation between disruptions in gut microbiota and primary membranous nephropathy (pMN), giving rise to the concept of the ‘gut-kidney axis’. However, the precise relationship between gut microbiota and pMN remains elusive. Hence, this study endeavors to investigate whether a causal relationship exists between gut microbiota and pMN utilizing Mendelian randomization (MR) analysis.

**Methods:**

The primary method employed for MR analysis is the inverse variance weighting method, supplemented by MR-Egger and the weighted median method, to infer causality. This approach was validated within the pMN cohort across two distinct populations.

**Results:**

At the species level, the abundance of *Bifidobacterium bifidum* and *Alistipes indistinctus* was negatively correlated with the risk of pMN. Conversely, pMN was positively associated with *Bacilli* abundance at the class level, *Lachnospiraceae* abundance at the family level, and *Dialister* abundance at the genus level. Specifically, at the species level, pMN was positively correlated with the abundance of *Ruminococcus lactaris*, *Dialister invisus*, and *Coprococcus_sp_ART55_1.*

**Conclusion:**

These findings lay the groundwork for future research exploring the interplay between pMN and the gut microbiota, with substantial implications for the prevention and treatment of pMN and its associated complications.

## Introduction

Membranous nephropathy (MN) is a prevalent form of primary nephrotic syndrome in adults [1]. Approximately 20% of patients are associated with secondary factors, including systemic diseases, infections, drugs, or tumors. Patients without identifiable secondary causes are classified as having primary membranous nephropathy (pMN) [2]. The onset of pMN is frequently observed in patients over 40 years old, with a gender ratio of about 2:1 male to female [1]. pMN is an autoimmune disease characterized by the activation of the body’s immune system by various podocyte antigens. The clinical progression of the disease is typically slow, with some patients experiencing spontaneous remission, while around 30% to 40% of individuals develop end-stage renal disease requiring dialysis or transplantation, or succumb [3]. Hence, there is an urgent need for research into the precise mechanism of pMN. Current studies suggest that dysregulation of gut ecology may play a crucial role in the pathophysiology of chronic kidney disease (CKD) [4].

An increasing body of research has established a connection between gut microbiota and the development of immunological disorders such as systemic lupus erythematosus and tumors [5,6]. To elucidate the relationship between the gut and the kidney, researchers have proposed the theory of the ‘gut-kidney axis’, suggesting that abnormalities in gut microbiota are associated with chronic renal diseases and may have a causal interaction [7]. Noninvasive diagnostic tools have been developed by analyzing the composition of intestinal microbiota, corresponding to different disease stages [8]. Recent studies have linked disruptions in gut microbiota to pMN [9], a connection also observed in animal models of pMN, where modulation of gut microbiota can ameliorate the disease [10]. It is also believed that gut microbiota analysis can be developed as a noninvasive diagnostic tool for pMN. A case report has documented improved renal function in pMN patients following fecal microbiota transplantation (FMT) [11], suggesting a significant impact of gut microbiota on pMN formation. However, the causal connection between pMN and gut microbiota remains unknown.

Mendelian randomization (MR) is a method of causal inference based on genetic variations, which examines how randomly assigned genotypes affect natural phenotypes to infer the impact of biological causes on diseases. MR offers distinct advantages over typical observational research by effectively mitigating the influence of confounding variables and reverse causality, thereby enhancing the reliability of results [12]. In this study, we were granted unrestricted access to genome-wide association study (GWAS) data summaries associated with primary membranous nephropathy (pMN), accessible through the Kiryluk Lab. We utilized gut microbiota-related information derived from the latest Dutch Microbiome Program GWAS data. This enabled us to perform a two-sample bidirectional MR analysis with the primary aim of investigating the causal relationship between gut microbiota and pMN.

## Materials and methods

### Research design and data sources

**Figure 1.**
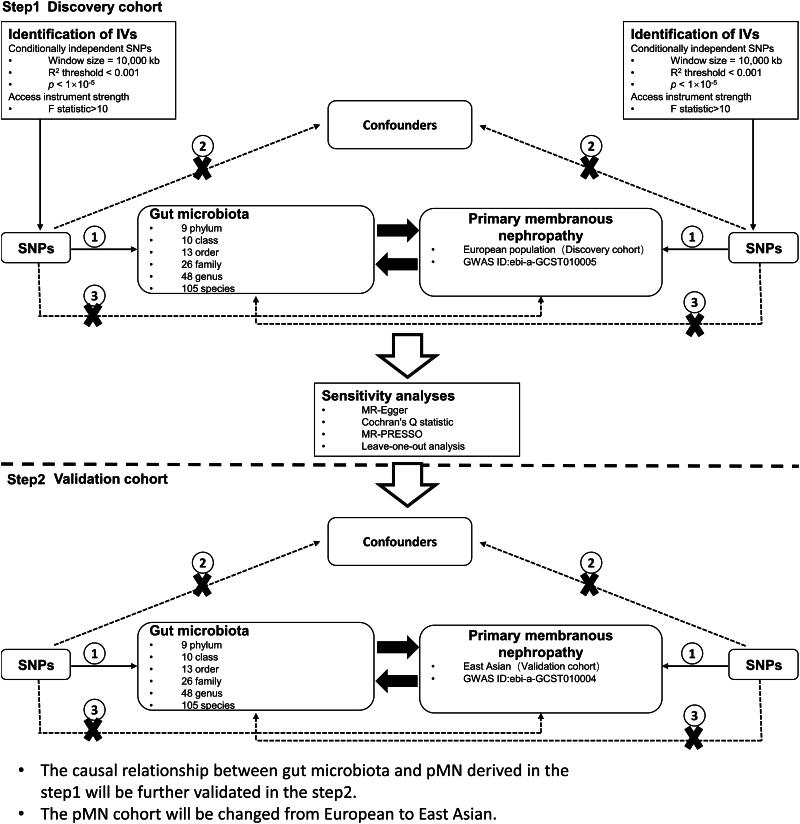
Study design for a two-sample bidirectional Mendelian randomization study on gut microbiota and primary membranous nephropathy (pMN). IV: instrumental variable; GWAS: genome-wide association study; SNP: single-nucleotide polymorphism; MR-PRESSO: Mendelian randomization polytropic residuals and outliers.

The study’s design overview and Mendelian randomization (MR) study assumptions are depicted in Figure 1. In MR research, instrumental variables (IVs) in the form of single nucleotide polymorphisms (SNPs) must satisfy three fundamental assumptions. Hypothesis 1 posits that genetic variations proposed as IVs should be strongly associated with the risk factors of interest. Hypothesis 2 states that the genetic variability utilized should not be correlated with potential confounding factors. Hypothesis 3 suggests that the selected genetic variation will exert its effect solely on the outcome through risk factors and not through any alternative pathways.

The genetic variation data related to primary membranous nephropathy (pMN) were acquired from the Kiryluk Lab [13], and are freely accessible through the IEU OPEN GWAS project. This summary of GWAS data was divided into two cohorts. The first cohort, identified by GWAS ID ebi-a-GCST010005, comprised a European population of 7979 individuals, including 2150 in the experimental group and 5829 in the control group, with a total of 5,327,688 single nucleotide polymorphisms (SNPs). This cohort was utilized as the discovery cohort in our study. The second cohort, designated by GWAS ID ebi-a-GCST010004, was composed of an East Asian population and was also utilized as the discovery cohort in our study. The GCST010004 cohort comprised 4841 East Asian participants, of whom 1632 were in the experimental group and 3209 were in the control group. A total of 3,877,927 SNPs were used as a validation cohort in this study.

Genome-wide association study (GWAS) data on gut microbiota were summarized from the Dutch Microbiome Project (DMP) [14]. The study population consisted of individuals residing in the northern part of the Netherlands, with 7738 DMP participants. Among them, 58.1% were female, and their ages ranged from 8 to 84 years, with a mean age of 48.5 years. The mean BMI was 25.58, ranging from 13.10 to 63.70. Summaries of GWAS data on gut microbiota abundance for 207 taxa, including 5 phyla, 10 classes, 13 orders, 26 families, 48 genera, and 105 species, at 6 taxonomic levels were obtained using Birdshot macrogenomic sequencing.

Further details regarding genome-wide association studies (GWAS) associated with pMN and gut microbiota can be found in Supplementary Table S1. This study did not require ethical approval as it utilized publicly available data and had previously obtained approval from the appropriate ethical review boards for all included GWAS data.

### Screening for instrumental variable SNPs

This article adheres to the requirements of STROBE-MR (Strengthening the Reporting of Observational Studies in Epidemiology) [15]. Supplementary Table S3 contains a list of STROBE-MR-recommended items. Two-sample Mendelian randomization (MR) analyses were conducted using the TwoSampleMR software package v.0.5.7. A less stringent significance level of *p* < 1 × 10^−5 was applied for genomic variations associated with each microbial exposure. In a previous study, this threshold was identified as optimal for identifying genetic predictors that explain a larger proportion of variance in the outcomes. The clump_data() method from the TwoSampleMR package was employed to aggregate significant single nucleotide polymorphisms (SNPs) and assess the independence of genetic variants. Linkage disequilibrium was mitigated by setting the R^2 threshold to be greater than 0.001, with a clumping window of 10,000 kb. To eliminate weak instrumental variables, the F-statistics threshold was set to be greater than 10, with the exact formula detailed in Supplementary Table S2 [16]. PhenoScanner, an online platform, was utilized by our team to identify and mitigate potential confounding factors associated with SNPs that could interfere with exposure-outcome associations. This SNP screening process enhanced the robustness and reliability of our findings.

### Statistical analysis

The inverse variance weighted (IVW) approach employed in this study is considered the primary analytical method [17]. It utilizes regressions that exclude intercept variables and are fitted with weights equal to the inverse of the variance of the outcomes. When there is no evidence of heterogeneity in level polytropy, the IVW fixed-effects model is utilized. Additionally, the weighted median approach was employed to bolster the aforementioned findings. The weighted median technique defines the outcome as the weighted median of the ratio estimates, enabling causal inference. This method requires that at least 50% of the information for the analysis originates from reliable instruments.

Sensitivity analyses were conducted using various tools. Firstly, Cochran’s Q test was applied to assess heterogeneity among individual SNP estimates. Significant heterogeneity was indicated if Cochran’s Q test yielded a statistically significant result. Secondly, prior to re-running the study, the Mendelian randomization pleiotropy residual sum and outlier (MR-PRESSO) approach were utilized to validate the IVW model findings and to identify and address any existing outliers. Thirdly, the MR-Egger intercept test was employed to investigate the potential horizontal pleiotropy of single nucleotide polymorphisms (SNPs). The presence of horizontal pleiotropy in the MR analysis was indicated by a substantial intercept term in the MR-Egger intercept test. Fourthly, leave-one-out sensitivity analyses were performed, involving the removal of one SNP at a time to evaluate if any variation could alter the relationship between exposure and outcome variables. Fifthly, funnel plots and forest plots were generated in our MR analysis to visualize and detect any potential horizontal pleiotropy. Statistical significance (*p* value < .05) suggests a possible cause-and-effect relationship in the MR analyses. All statistical examinations were conducted using the ‘TwoSampleMR’ package in R software version 4.3.1.

## Result

### Screening for SNPs

In this investigation, a two-sample two-way Mendelian randomization (MR) analysis was conducted. Two distinct cohorts of primary membranous nephropathy (pMN) from European and East Asian populations were utilized. During the forward MR analysis on the discovery cohort, gut microbiota was designated as the exposure factor. A total of 207 categorical gut microbiota instrumental variables were screened, and the pMN cohort of the European population was set as the outcome variable and subsequently analyzed. For the reverse MR analysis, instrumental variables were screened for the pMN cohort of the European population, with pMN as the exposure factor and gut flora as the outcome variable.

To validate the results in the discovery cohort, we utilized gut microbiota with a *p* value < .05 obtained from the IVW analysis in the forward analysis of the discovery cohort as an exposure factor during the forward analysis in the validation cohort. Subsequently, we screened for relevant SNPs and defined the pMN of the East Asian population as the outcome variable. In a reverse analysis, SNPs from the pMN cohort of the East Asian population were screened to identify pMN as an exposure factor. The microbiota with *p* < .05, determined from IVW analysis of the discovery cohort, was then validated as an outcome variable.

The F-statistics of the instrumental variables (IVs) in our analysis all exceed 10, indicating the absence of weak instrumental variables. Further details on the selected instrumental variables and harmonized data can be found in Supplementary Table S2.

### Discovery cohort bidirectional MR and sensitivity analysis

**Figure 2.**
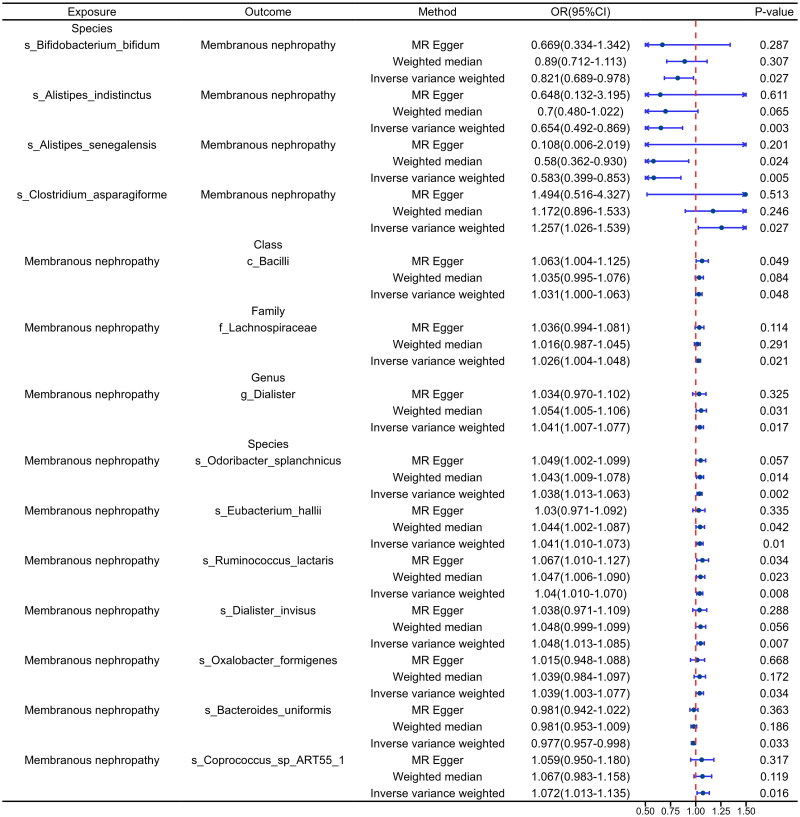
Discovery cohort MR analysis of the main results. The exposure factor in the forward analysis was gut microbiota and in the reverse analysis was primary membranous nephropathy. OR: odds ratio; CI: confidence interval.

In the analysis of the discovery cohort, gut microbiota was examined as an exposure factor, revealing a species-level causal association between four gut microbiota and pMN (Figure 2). Notably, *s_Bifidobacterium_bifidum* (Odds Ratio (OR): 0.821, 95% Confidence Interval (CI): 0.69–0.978, *p* = .0272), *s_Alistipes_indistinctus* (OR: 0.654, 95% CI: 0.492–0.869, *p* = .0034), and *s_Alistipes_senegalensis* (OR: 0.583, 95% CI: 0.399–0.853, *p* = .0054) were identified as protective factors against pMN. Conversely, *s_Clostridium_asparagiforme* (OR: 1.257, 95% CI: 1.026–1.539, *p* = .0271) was significantly associated with an increased risk of pMN. Sensitivity analyses conducted with MR-Egger, Heterogeneity, and MR-PRESSO revealed no evidence of heterogeneity or pleiotropy (*p* > .05) (Table 1). Leave-one-out testing consistently showed similar results regardless of the SNP removed (Supplementary Figure S1A). Additionally, forest plots, scatter plots, and funnel plots exhibited consistent findings (Supplementary Figures S2A, S3A, and S4A).

**Figure 3.**
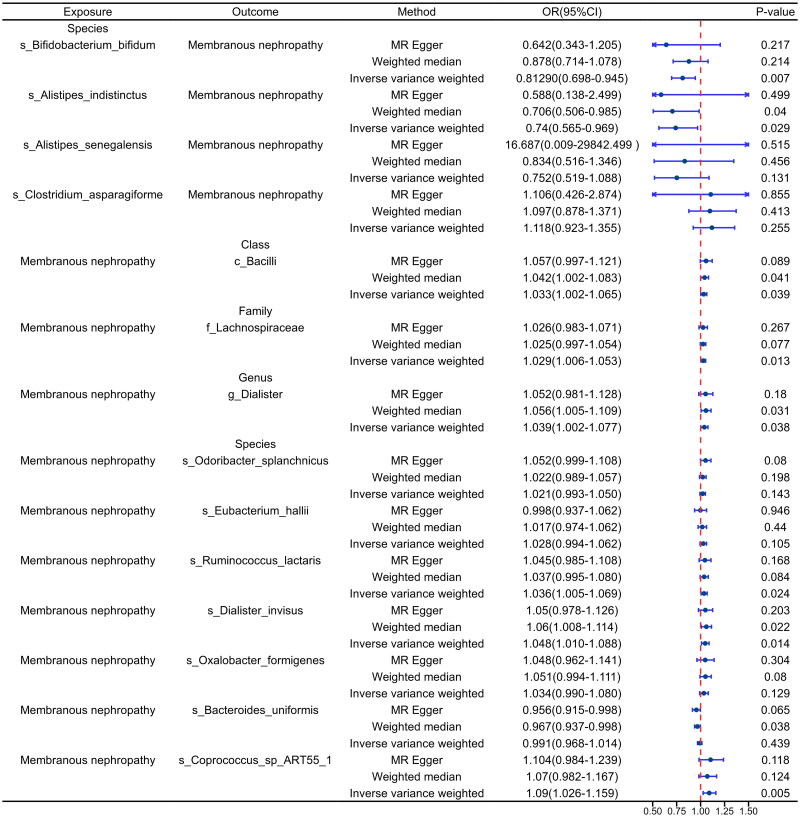
Validation of the main results of the cohort MR analysis. The exposure factor in the forward analysis was gut microbiota and in the reverse analysis was primary membranous nephropathy. OR: odds ratio; CI: confidence interval.

**Table 1.** Discovery cohort sensitivity analysis of the main results.

Exposure	Outcome	MR-Egger		Heterogeneity		MR-PRESSO	
		Intercept	*p*	*Q*-value	*p*	RSSOBs	*p*
Species							
* s_Bifidobacterium_bifidum*	Membranous nephropathy	0.0395	.5659	12.1598	.2045	22.83102	.091
* s_Alistipes_indistinctus*		0.0011	.9914	4.7171	.6944	6.6625	.8234
* s_Alistipes_senegalensis*		0.1612	.3114	0.202	.999	5.682	.7468
* s_Clostridium_asparagiforme*		−0.0432	.7667	2.537	.4686	11.0502	.3222
	Class						
Membranous nephropathy	c_Bacilli	−0.0143	.2309	19.5597	.3581	28.5617	.2654
	Family						
Membranous nephropathy	f_Lachnospiraceae	−0.0048	.5832	19.6669	.3519	22.6065	.574
	Genus						
Membranous nephropathy	g_Dialister	0.0035	.7907	17.897	.4625	22.8974	.5496
	Species						
Membranous nephropathy	s_Odoribacter_splanchnicus	−0.005	.602	15.6679	.6157	18.9534	.762
	s_Eubacterium_hallii	0.0049	.6836	14.2044	.7157	19.1666	.7608
	s_Ruminococcus_lactaris	−0.012	.2964	17.578	.4837	22.9297	.5538
	s_Dialister_invisus	0.0048	.7233	14.6394	.6866	18.3883	.7844
	s_Oxalobacter_formigenes	0.0111	.4525	18.1884	.377	25.7236	.3516
	s_Bacteroides_uniformis	−0.0017	.8361	11.7428	.8602	21.9496	.6084
	s_Coprococcus_sp_ART55_1	0.0059	.7928	15.3027	.6411	19.6504	.7306

*Note:* MR-PRESSO: Mendelian randomization pleiotropy residual sum and outlier; *p*: *p* value; Q: Cochran’s Q test.

When analyzing pMN as a reversal of exposure, causality between pMN and 10 gut microbiota was established (Figure 2). Positive correlations were observed between pMN and *c_Bacilli* (OR: 1.031, 95% CI: 1.00–1.06, *p* = .048) at the class level, *f_Lachnospiraceae* (OR: 1.026, 95% CI: 1.004–1.048, *p* = .0271) at the family level, and *g_Dialister* (OR: 1.041, 95% CI: 1.007–1.077, *p* = .017) at the genus level. pMN was shown to be causally related to seven different types of gut microbiota at the species level. Among them, pMN was associated with *s_Odoribacter_splanchnicus* (OR: 1.038, 95% CI: 1.013–1.063, *p* = .002), *s_Eubacterium_hallii* (OR: 1.041, 95% CI: 1.010–1.073, *p* = .01), *s_Ruminococcus_lactaris* (OR: 1.04, 95% CI: 1.010–1.070, *p* = .008), *s_Dialister_invisus* (OR: 1.048, 95% CI: 1.013–1.085, *p* = .007), *s_Oxalobacter_formigenes* (OR: 1.039, 95% CI: 1.003–1.077, *p* = .034), and *s_Coprococcus_sp_ART55_1* (OR: 1.072, 95% CI: 1.013–1.135, *p* = .016). Additionally, *s_Bacteroides_uniformis* (OR: 0.977, 95% CI: 0.957–0.998, *p* = .033) was negatively correlated.

Consistent with the findings from the forward analysis, MR-Egger, Heterogeneity, and MR-PRESSO results from the reverse analysis indicated a lack of heterogeneity and pleiotropy (*p* > .05) (Table 1). Leave-one-out tests demonstrated that the overall results remained stable after the exclusion of any individual SNP (Supplementary Figure S1 B-C). Similar results were observed for forest plots, scatter plots, and funnel plots (Supplementary Figure S2 B-C, Supplementary Figure S3 B-C, and Supplementary Figure S4 B-C). Complete details of the leave-one-out analysis, forest plot, scatter plot, and funnel plot for the current dataset are available in Supplementary Figures S1–S4.

### Validation of queue bidirectional MR and sensitivity analysis

To validate the 14 causal relationships identified in the discovery cohort, we utilized the pMN cohort (GWAS ID: ebi-a-GCST010004) from the East Asian population as the validation cohort. In the analysis where gut microbiota served as the exposure, we identified two microbiota with causal relationships with pMN. Specifically, *s_Bifidobacterium_bifidum* (OR: 0.8129, 95% CI: 0.698–0.945, *p* = .007) and *s_Alistipes_indistinctus* (OR: 0.74, 95% CI: 0.565–0.969, *p* = .029) were identified as protective factors for pMN (Figure 3).

In the reverse analysis where pMN was treated as the exposure, we confirmed six causal relationships (Figure 3). At the class level, pMN exhibited a positive correlation with the abundance of *c_Bacilli* (OR: 1.033, 95% CI: 1.002–1.065, *p* = .039), at the family level, with the abundance of *f_Lachnospiraceae* (OR: 1.029, 95% CI: 1.006–1.053, *p* = .013), and at the genus level, with the abundance of *g_Dialister* (OR: 1.039, 95% CI: 1.002–1.077, *p* = .038). At the species level, pMN was positively associated with increased abundance of *s_Ruminococcus_lactaris* (OR: 1.036, 95% CI: 1.005–1.069, *p* = .024), *s_Dialister_invisus* (OR: 1.048, 95% CI: 1.010–1.088, *p* = .014), and *s_Coprococcus_sp_ART55_1* (OR: 1.09, 95% CI: 1.026–1.159, *p* = .005).

Validation results from MR-Egger, Heterogeneity, and MR-PRESSO indicated the absence of heterogeneity and pleiotropy (*p* > .05), as presented in Table 2. Leave-one-out tests demonstrated consistent overall findings even after removing individual SNPs (Supplementary Figure S5 B-C). Similar results were observed for forest plots, scatter plots, and funnel plots (Supplementary Figure S6 B-C, Supplementary Figure S7 B-C, and Supplementary Figure S8 B-C). Detailed information on leave-one-out, forest plot, scatter plot, and funnel plot for the validation cohort can be found in Supplementary Figures S5 to S8.

**Table 2.** Validation cohort sensitivity analysis of the main results.

Exposure	Outcome	MR-Egger		Heterogeneity		MR-PRESSO	
		Intercept	*p*	*Q*-value	*p*	RSSOBs	*p*
Species							
* s_Bifidobacterium_bifidum*	Membranous nephropathy	0.0465	.4788	6.7139	.3481	14.9406	.213
* s_Alistipes_indistinctus*		0.028	.7609	8.6945	.1915	11.9385	.3372
* s_Alistipes_senegalensis*		−0.2853	.4761	1.4694	.6893	5.6071	.587
* s_Clostridium_asparagiforme*		0.0026	.984	0.6405	.726	6.0708	.534
	Class						
Membranous nephropathy	c_Bacilli	−0.0112	.3845	12.1467	.434	19.7205	.4376
	Family						
Membranous nephropathy	f_Lachnospiraceae	0.0016	.8594	8.0328	.7104	10.7004	.8932
	Genus						
Membranous nephropathy	g_Dialister	−0.0062	.6827	11.5482	.3985	20.3026	.3722
	Species						
Membranous nephropathy	s_Odoribacter_splanchnicus	−0.0143	.2139	13.2987	.3477	19.1047	.4674
	s_Eubacterium_hallii	0.0144	.2965	9.4404	.6649	21.8398	.3216
	s_Ruminococcus_lactaris	0.004	.7535	8.039	.7821	10.5903	.9282
	s_Dialister_invisus	−0.0008	.9602	10.3281	.5011	18.8024	.4484
	s_Oxalobacter_formigenes	−0.0063	.7316	17.4232	.1344	27.9275	.1506
	s_Bacteroides_uniformis	0.0177	.0798	7.7871	.7322	18.08083	.5318
	s_Coprococcus_sp_ART55_1	−0.006	.8037	11.4513	.4907	16.43433	.6466

*Note:* MR-PRESSO: Mendelian randomization pleiotropy residual sum and outlier; *p*: *p* value; *Q*: Cochran’s Q test value.

## Discussion

This two-sample MR study showed that the abundance of *Bifidobacterium bifidum* and *Alistipes indistinctus* at the species level was negatively correlated with the risk of pMN. pMN showed a positive correlation with *Bacilli* at the class level, *Lachnospiraceae* at the family level, and *Dialister* at the genus level. At the same time, pMN is positively correlated with the abundance of *Ruminococcus lactaris*, *Dialister invisus*, and *Coprocccus _ SP _ ART55 _ 1* at the species level. This has significant implications for potentially preventing and treating pMN and its related complications.

The gut microbiota undergoes structural and functional changes throughout different stages of the human life cycle, and its relation to human health and disease development has been established [18]. As previously mentioned, pMN is also strongly associated with the development of gut microbiota. *Bifidobacterium*, the prominent intestinal probiotic, is abundant in the adult intestinal tract. Lang et al.'s 16S rRNA sequencing analysis of fecal samples collected from pMN patients indicated a reduction in the abundance of *Bifidobacterium* [19]. Similarly, a study by Miao et al. found that *Bifidobacterium* as a probiotic was reduced in abundance in patients with pMN and verified this result in a further animal model, where the AhR signaling pathway may be involved in the process of action [10]. Interestingly, in patients with CKD, renal function was improved after supplementation with probiotics of the patient’s own origin, such as *Bifidobacterium* [20].

Immunoglobulin A (IgA) plays a crucial role in maintaining mucosal homeostasis and defending against pathogens, with secretory IgA (SIgA) being capable of aggregating pathogens, regulating intestinal bacterial colonization, and being influenced by the gut microbiota [21]. Studies have found that *Bifidobacterium* can increase levels of SIgA while reducing NF-κB expression, aiding in lowering levels of lipopolysaccharides, IL-1β, and IL-6 [22]. In mice fed a high-protein diet, there was a significant increase in *Bifidobacterium* abundance, which could modulate the cytokine environment in the intestinal lamina propria [23]. The primary mechanism involved an increase in April expression, promoting the elevation of SIgA levels. These findings indicate that dysregulation of Bifidobacterium is linked to the development of pMN, which is consistent with the results of our two-sample MR analysis.

*Alistipes* is a gram-negative bacterium belonging to the Mycobacterium phylum. Ongoing research indicates a disrupted ecological balance of *Alistipes*, which can prove advantageous or detrimental. Specifically, *Alistipes* proved to be beneficial in investigations concerning liver fibrosis, cancer immunotherapy, and cardiovascular disease [24–26]. On the other hand, previous findings have demonstrated the association of *Alistipes* with depression and its pathogenic role in colorectal cancer [27,28]. The impact of *Alistipes* on the secretion levels of SIgA in the intestine remains unknown. Only in ulcerative colitis mouse models has an increase in *Alistipes* abundance been observed, accompanied by a significant decrease in SIgA secretion levels. However, further research is needed to determine whether there is a correlation between the two [29]. So far, there is no definitive study showing how the abundance of Alistipes changes in pMN. However, our MR study indicated a potential protective effect of *Alistipes* indistinctus against pMN, although additional investigations are necessary to substantiate this finding.

Reverse MR studies confirmed the association of pMN with six gut microbiota. At the class level, pMN showed a positive correlation with *Bacilli*. However, limited research exists regarding Bacilli’s role in pMN, with only one case report describing *Bacilli* infection leading to pMN development [30]. Conversely, in animal models, Bacilli were found to inhibit the TLR4/MyD88/NF-κB pathway, reduce various inflammatory factors’ expression, and upregulate SIgA expression in the small intestinal mucosa [31]. Thus, *Bacilli’s* specific role in pMN warrants validation in large-scale human cohorts and relevant animal models. At the family level, pMN exhibited a positive correlation with *Lachnospiraceae*, contrary to Xiang et al.'s findings. This discrepancy may stem from population and sample size differences or variations in disease stage [32]. Additionally, potential confounding factors may exist, leading to opposing results. Recent studies have shown that *Lachnospiraceae’s* high enrichment in the intestine can increase SIgA secretion [33], possibly reflecting a negative feedback mechanism in response to the microbiota. pMN is positively associated with *Citrobacter* at the genus level, despite the lack of documented studies in pMN. Previous findings suggest that this bacterial group’s intestinal colonization can activate IL-17 signaling, leading to pathogen-specific IgA secretion [34]. Hence, pMN may increase *Citrobacter* abundance, promoting intestinal SIgA secretion. The study results indicate pMN can increase *Ruminococcus* abundance at the genus level, aligning with previous findings of higher *Ruminococcus* abundance in adult pMN compared to healthy controls [35]. *Ruminococcus* is generally considered pathogenic, with its abundance negatively correlated with intestinal SIgA levels [36]. Therefore, more attention to changes in *Ruminococcus* abundance is needed for the prevention and treatment of pMN. Therefore, monitoring *Ruminococcus* abundance is crucial for pMN prevention and treatment. *Dialister invisus* has limited discussion in pMN research, although recent studies link it to fasting glucose levels in diabetic renal transplant patients and its dominance in CKD [37,38]. Its impact on intestinal SIgA remains unclear, necessitating further exploration. Finally, our study shows *Coprococcus_sp_ART55_1* abundance positively correlates with pMN, suggesting the pMN environment may increase its abundance. However, the specific mechanism in pMN remains unclear [19].

As extensively noted, gut microbiota dysbiosis is prevalent among pMN patients. However, this dysbiosis may merely reflect a clinical manifestation of pMN, given the unknown link between pMN and gut flora. Several factors contribute to this inference. Firstly, the frequent use of glucocorticoids and immunosuppressive drugs among pMN patients may independently induce alterations in gut flora [39,40]. Secondly, patients often experience varying degrees of gut microbiota alterations at different disease progression stages, but many studies have failed to adequately account for these factors in protocol design [41]. Thirdly, differences in geography, race, gender, and dietary culture among various pMN cohort studies can all impact gut microbiota changes. Lastly, previous studies have indicated varying levels of dysbiosis in pMN, yet inconsistency in specific strain variations has been observed, making it challenging to establish a causal association between pMN and gut microbiota due to these uncertainties.

In this study, the discovery and validation cohorts for pMN GWAS data were sourced from different populations. Specifically, the discovery cohort originated from a European population, while the validation cohort was obtained from an East Asian population. In recent years, mounting evidence has highlighted significant differences in gut microbiota composition among various ethnicities [42]. These disparities manifest in diverse dietary habits, genetic backgrounds, and living environments, all of which are pivotal in understanding human health and disease occurrence [43]. Notably, substantial diversity in gut microbiota diversity is observed across different populations. Some studies indicate that populations in Africa and South America exhibit higher microbiota diversity in the gut, while those in Europe and North America demonstrate relatively lower diversity [43]. These variations may be closely tied to diverse dietary patterns, lifestyles, and environmental factors across different regions [43,44]. Additionally, disparities in gut microbiota composition are evident among different populations. For instance, certain beneficial bacterial genera such as *Bifidobacterium* and *Lactobacillus* are more abundant in the gut microbiota of Asian individuals, whereas Western populations tend to harbor more Bacteroides and other genera [42,43,45]. Substantial dietary differences among populations may be a primary factor contributing to these variations in gut microbiota [46,47]. For example, traditional Asian diets primarily consist of rice, vegetables, and soybeans, while Western diets often feature high-fat and high-protein foods. These dietary disparities may lead to differences in gut microbiota composition, thereby impacting the host’s health status [48]. Besides dietary and lifestyle factors, genetic factors may also contribute to inter-population differences in gut microbiota [49]. Some studies suggest that individual genetic backgrounds influence gut microbiota composition. Furthermore, environmental factors such as living conditions and hygiene practices may also influence variations in gut microbiota composition [50]. These factors interact with each other, collectively influencing gut microbiota diversity and composition. Despite the multifaceted influences on gut microbiota among different populations, stable and consistent bacterial characteristics still exist in the gut [51]. Therefore, employing validation cohorts from different populations remains feasible in our MR study.

The MR analysis employed in this study offers significant advantages. Firstly, it effectively addresses confounding factors and reverse causality, thereby enhancing the credibility of the study findings. Secondly, the pMN cohort was assessed using both European and East Asian populations, which strengthens the reliability of the results. Additionally, the GWAS data on gut microbiota abundance were generated using macrogenomic birdshot sequencing, a more reliable method than traditional 16S rRNA sequencing, capable of providing accurate species-level data [52]. However, several limitations should be acknowledged. Although a pleiotropy test was conducted, potential pleiotropy cannot be completely ruled out due to the nature of the MR design. It remains challenging to entirely eliminate the influence of confounders during confounder exclusion. Furthermore, while our study included two distinct populations in the pMN cohort, the GWAS data for gut microbiota were obtained solely from European populations, which may have impacted our findings.

## Conclusion

The results of the bidirectional MR analysis showed a negative causal association between *Bifidobacterium bifidum* and *Alistipes indistinctus* in the gut microbiota and pMN. On the other hand, pMN exhibited a positive causal association with the abundance of *Bacilli* at the class level and *Lachnospiraceae* at the family level. Additionally, the gut microbiota abundance of Genus *Dialister* at the class level was positively causally linked to pMN. Additionally, pMN demonstrated a positive correlation with the abundance of *Ruminococcus lactaris*, *Dialister invisus*, and *Coprococcus_sp_ART55_1* at the species level. This finding holds significance for potential investigations studying pMN in combination with gut microbiota-related studies, providing a foundation for the prevention and treatment of pMN and associated complications.

## Supplementary Material

Supplemental Material

## Data Availability

All the original GWAS data used in this study can be obtained free of charge in the IEU OPEN GWAS project. The website is https://gwas.mrcieu.ac.uk/.
