# Supplementary material for: Evidence from mendelian randomization identifies several causal relationships between primary membranous nephropathy and gut microbiota
Source: Ren Fail. 2024 May 21;46(1):2349136. doi: 10.1080/0886022X.2024.2349136 (PMC11110878; doi:10.1080/0886022X.2024.2349136)
Supplement: Supplemental Material [file IRNF_A_2349136_SM1759.zip › table S3.docx]

**STROBE-MR checklist of recommended items to address in reports of Mendelian randomization studies**^1^ ^2^

| **Item No.** | **Section** | **Checklist item** | **Page No.** | **Relevant text from manuscript** |
| --- | --- | --- | --- | --- |
| 1 | **TITLE and ABSTRACT** | Indicate Mendelian randomization (MR) as the study’s design in the title and/or the abstract if that is a main purpose of the study | 1–3 | Complete |
|  | **INTRODUCTION** |  |  |  |
| 2 | **Background** | Explain the scientific background and rationale for the reported study. What is the exposure? Is a potential causal relationship between exposure and outcome plausible? Justify why MR is a helpful method to address the study question | 5–7 | Complete –The Introduction provides the scientific background and specific requirements for Mendelian randomization regarding the gut microbiota and primary membranous nephropathy. These details are presented in paragraphs 1, 2, and 3. |
| 3 | **Objectives** | State specific objectives clearly, including pre-specified causal hypotheses (if any). State that MR is a method that, under specific assumptions, intends to estimate causal effects | 6–7 | Complete – Objectives has been clarified in the paragraph 3 of the introduction. |
|  | **METHODS** |  |  |  |
| 4 | **Study design and data sources** | Present key elements of the study design early in the article. Consider including a table listing sources of data for all phases of the study. For each data source contributing to the analysis, describe the following: |  |  |
|  | a) | Setting: Describe the study design and the underlying population, if possible. Describe the setting, locations, and relevant dates, including periods of recruitment, exposure, follow-up, and data collection, when available. | 8-9 | Complete – All necessary information about the study design and underlying population has been described in the Material and methods section “Research design and data sources” and Supplementary table S1 |
|  | b) | Participants: Give the eligibility criteria, and the sources and methods of selection of participants. Report the sample size, and whether any power or sample size calculations were carried out prior to the main analysis | 8-9 | Complete – All necessary information about GWAS studies been used in this study has been described in Material and methods section and Supplementary table S1. |
|  | c) | Describe measurement, quality control and selection of genetic variants | 10 | Complete – The genetic predictor selection process has been described in the Material and methods section “2.2 Screening for instrumental variable SNPs”. |
|  | d) | For each exposure, outcome, and other relevant variables, describe methods of assessment and diagnostic criteria for diseases | 8 | Complete – The information about the diagnostic criteria for diseases has been described in the Material and methods section. |
|  | e) | Provide details of ethics committee approval and participant informed consent, if relevant | 8 | Complete – Ethical approval and participant informed consent has been described in the Material and methods section. |
| 5 | **Assumptions** | Explicitly state the three core IV assumptions for the main analysis (relevance, independence and exclusion restriction) as well assumptions for any additional or sensitivity analysis | 10-11 | Complete – The Mendelian randomization assumptions have been described in the Material and methods section “2.1 Research design and data sources” and Figure 1. |
| 6 | **Statistical methods: main analysis** | Describe statistical methods and statistics used |  |  |
|  | a) | Describe how quantitative variables were handled in the analyses (i.e., scale, units, model) |  | NA |
|  | b) | Describe how genetic variants were handled in the analyses and, if applicable, how their weights were selected | 10-11 | Complete – Described in the “2.2 Screening for instrumental variable SNPs” and “2.3 Statistical analysis” of the Material and methods section. |
|  | c) | Describe the MR estimator (e.g. two-stage least squares, Wald ratio) and related statistics. Detail the included covariates and, in case of two-sample MR, whether the same covariate set was used for adjustment in the two samples | 10-11 | Complete – Described in the “2.3 Statistical analysis” of the Material and methods section. |
|  | d) | Explain how missing data were addressed |  | NA |
|  | e) | If applicable, indicate how multiple testing was addressed |  | NA |
| 7 | **Assessment of assumptions** | Describe any methods or prior knowledge used to assess the assumptions or justify their validity | 10-11 | Complete – Described in the “2.3.2 Statistical Analysis and MR Assumptions” of the Material and methods section. |
| 8 | **Sensitivity analyses and additional analyses** | Describe any sensitivity analyses or additional analyses performed (e.g. comparison of effect estimates from different approaches, independent replication, bias analytic techniques, validation of instruments, simulations) | 10-12 | Complete – All necessary information about additional analyses and sensitivity analyses are described in “2.3 Statistical analysis” of the Material and methods section. |
| 9 | **Software and pre-registration** |  |  |  |
|  | a) | Name statistical software and package(s), including version and settings used | 10 | Complete – The package and settings used are described in the Material and methods section. |
|  | b) | State whether the study protocol and details were pre-registered (as well as when and where) |  | NA |
|  | **RESULTS** |  |  |  |
| 10 | **Descriptive data** |  |  |  |
|  | a) | Report the numbers of individuals at each stage of included studies and reasons for exclusion. Consider use of a flow diagram |  | Complete – See Figure 1 and Supplementary table S1. |
|  | b) | Report summary statistics for phenotypic exposure(s), outcome(s), and other relevant variables (e.g. means, SDs, proportions) |  | Complete – See Figure 2 to 3 and Table 1 to 2. |
|  | c) | If the data sources include meta-analyses of previous studies, provide the assessments of heterogeneity across these studies | 14 | NA |
|  | d) | For two-sample MR:  i.  Provide justification of the similarity of the genetic variant-exposure associations between the exposure and outcome samples  ii.  Provide information on the number of individuals who overlap between the exposure and outcome studies | 11 | Complete – The information on the number of individuals who overlap between the exposure and outcome studies are described in the Material and methods section. |
| 11 | **Main results** |  |  |  |
|  | a) | Report the associations between genetic variant and exposure, and between genetic variant and outcome, preferably on an interpretable scale |  | Complete – The associations have been reported in Supplementary table S2. |
|  | b) | Report MR estimates of the relationship between exposure and outcome, and the measures of uncertainty from the MR analysis, on an interpretable scale, such as odds ratio or relative risk per SD difference | 15 | Complete – MR estimates of the relationship between exposure and outcome are shown in Figure 2 to 3 and Table 1 to 2. |
|  | c) | If relevant, consider translating estimates of relative risk into absolute risk for a meaningful time period | 15 | Complete – Our results were presented in terms of odds ratio and confidence intervals throughout the results section for binary outcomes and as beta coefficient for quantitative outcomes. |
|  | d) | Consider plots to visualize results (e.g. forest plot, scatterplot of associations between genetic variants and outcome versus between genetic variants and exposure) |  | Complete – See Figure S1 to S8. |
| 12 | **Assessment of assumptions** |  |  |  |
|  | a) | Report the assessment of the validity of the assumptions | 15 | Complete – We assessed the validity using IVW, MR-Egger, weighted median, simple mode, and weighted mode, as shown in the Results section |
|  | b) | Report any additional statistics (e.g., assessments of heterogeneity across genetic variants, such as *I^2^*, Q statistic or E-value) | 16 | Complete – We reported the use of Cochran’s Q in the Results section. |
| 13 | **Sensitivity analyses and additional analyses** |  |  |  |
|  | a) | Report any sensitivity analyses to assess the robustness of the main results to violations of the assumptions | 15 | Complete – We complemented IVW with MR-Egger and weighte as sensitivity analyses. See Figure 2 to 3. |
|  | b) | Report results from other sensitivity analyses or additional analyses | 15-16 | Complete – We conducted MR-PRESSO global test, Cochran’s Q test, and leave-one-out analyses. See Table 2 to 3 and Supplementary Figure S1 to S8. |
|  | c) | Report any assessment of direction of causal relationship (e.g., bidirectional MR) | 15-16 | Complete –We performed two-way Mendelian randomization. Specific results are described in the results section |
|  | d) | When relevant, report and compare with estimates from non-MR analyses |  | NA |
|  | e) | Consider additional plots to visualize results (e.g., leave-one-out analyses) | 16 | Complete – See Figure S1 to S8. |
|  | **DISCUSSION** |  |  |  |
| 14 | **Key results** | Summarize key results with reference to study objectives | 18 | Complete – Discussion paragraph 1. |
| 15 | **Limitations** | Discuss limitations of the study, taking into account the validity of the IV assumptions, other sources of potential bias, and imprecision. Discuss both direction and magnitude of any potential bias and any efforts to address them | 20-21 | Complete – Discussion paragraph 5. |
| 16 | **Interpretation** |  |  |  |
|  | a) | Meaning: Give a cautious overall interpretation of results in the context of their limitations and in comparison with other studies | 18-19 | Complete – Discussion paragraph 4-5. |
|  | b) | Mechanism: Discuss underlying biological mechanisms that could drive a potential causal relationship between the investigated exposure and the outcome, and whether the gene-environment equivalence assumption is reasonable. Use causal language carefully, clarifying that IV estimates may provide causal effects only under certain assumptions | 19-20 | Complete – Discussion paragraph 2-3. |
|  | c) | Clinical relevance: Discuss whether the results have clinical or public policy relevance, and to what extent they inform effect sizes of possible interventions | 22 | Complete – Conclusions. |
| 17 | **Generalizability** | Discuss the generalizability of the study results (a) to other populations, (b) across other exposure periods/timings, and (c) across other levels of exposure | 20-21 | Complete – Discussion paragraph 5. |
|  | **OTHER INFORMATION** |  |  |  |
| 18 | **Funding** | Describe sources of funding and the role of funders in the present study and, if applicable, sources of funding for the databases and original study or studies on which the present study is based | 23 | Complete –See “Funding Statement” section. |
| 19 | **Data and data sharing** | Provide the data used to perform all analyses or report where and how the data can be accessed, and reference these sources in the article. Provide the statistical code needed to reproduce the results in the article, or report whether the code is publicly accessible and if so, where |  | Complete – The data used in the study can be accessed and downloaded from original studies. |
| 20 | **Conflicts of Interest** | All authors should declare all potential conflicts of interest | 24 | Complete – All authors declare that they have no conflict of interest. |

This checklist is copyrighted by the Equator Network under the Creative Commons Attribution 3.0 Unported (CC BY 3.0) license.

1. Skrivankova VW, Richmond RC, Woolf BAR, Yarmolinsky J, Davies NM, Swanson SA, et al. Strengthening the Reporting of Observational Studies in Epidemiology using Mendelian Randomization (STROBE-MR) Statement. JAMA. 2021;under review.

2. Skrivankova VW, Richmond RC, Woolf BAR, Davies NM, Swanson SA, VanderWeele TJ, et al. Strengthening the Reporting of Observational Studies in Epidemiology using Mendelian Randomisation (STROBE-MR): Explanation and Elaboration. BMJ. 2021;375:n2233.
